# Innate Immune Response against *Staphylococcus aureus* Preincubated with Subinhibitory Concentration of *trans*-Anethole

**DOI:** 10.3390/ijms21114178

**Published:** 2020-06-11

**Authors:** Paweł Kwiatkowski, Bartosz Wojciuk, Iwona Wojciechowska-Koszko, Łukasz Łopusiewicz, Bartłomiej Grygorcewicz, Agata Pruss, Monika Sienkiewicz, Karol Fijałkowski, Edward Kowalczyk, Barbara Dołęgowska

**Affiliations:** 1 Department of Diagnostic Immunology, Chair of Microbiology, Immunology and Laboratory Medicine, Pomeranian Medical University in Szczecin, 70-111 Szczecin, Poland; bartosz.wojciuk@pum.edu.pl (B.W.); iwonakoszko@interia.pl (I.W.-K.); 2 Center of Bioimmobilisation and Innovative Packaging Materials, Faculty of Food Sciences and Fisheries, West Pomeranian University of Technology Szczecin, 71-270 Szczecin, Poland; lukasz.lopusiewicz@zut.edu.pl; 3 Department of Laboratory Medicine, Chair of Microbiology, Immunology and Laboratory Medicine, Pomeranian Medical University in Szczecin, 70-111 Szczecin, Poland; bartlomiej.grygorcewicz@pum.edu.pl (B.G.); agata.pruss@pum.edu.pl (A.P.); barbara.dolegowska@pum.edu.pl (B.D.); 4 Department of Allergology and Respiratory Rehabilitation, Medical University of Łódź, 90-752 Łódź, Poland; monika.sienkiewicz@umed.lodz.pl; 5 Department of Microbiology and Biotechnology, Faculty of Biotechnology and Animal Husbandry, West Pomeranian University of Technology Szczecin, 70-311 Szczecin, Poland; karol.fijalkowski@zut.edu.pl; 6 Department of Pharmacology and Toxicology, Medical University of Łódź, 90-752 Łódź, Poland; edward.kowalczyk@umed.lodz.pl

**Keywords:** *trans*-anethole, staphyloxanthin, phagocytosis, IL-8, *Staphylococcus aureus*

## Abstract

The study aimed to analyze morphological and functional changes of *Staphylococcus aureus* cells due to *trans*-anethole (a terpenoid and the major constituent of fennel, anise, or star anise essential oils) exposition, and their consequences for human neutrophils phagocytic activity as well as IL-8 production (recognized as the major chemoattractant). The investigation included the evaluation of changes occurring in *S. aureus* cultures, i.e., staphyloxanthin production, antioxidant activities, cell size distribution, and cells composition as a result of incubation with *trans*-anethole. It was found that the presence of *trans*-anethole in the culture medium reduced the level of staphyloxanthin production, as well as decreased antioxidant activities. Furthermore, *trans*-anethole-treated cells were characterized by larger size and a tendency to diffuse in comparison to the non-treated cells. Several cell components, such as phospholipids and peptidoglycan, were found remarkably elevated in the cultures treated with *trans*-anethole. As a result of the aforementioned cellular changes, the bacteria were phagocytized by neutrophils more efficiently (ingestion and parameters associated with killing activity were at a higher level as compared to the control system). Additionally, IL-8 production was at a higher level for *trans*-anethole modified bacteria. Our results suggest that *trans*-anethole represents a promising measure in combating severe staphylococcal infections, which has an important translational potential for clinical applications.

## 1. Introduction

*Staphylococcus aureus* is recognized as an outstandingly significant human pathogen responsible for a spectrum of clinical conditions, including asymptomatic skin carriage, mild skin infections, severe systemic infections, and septic shock. Currently, *S. aureus* is characterized as an extracellular pathogen, and a strong inducer of phagocytosis and pyogenic reactions. Additionally, asymptomatic skin carriage and the potential to cause severe systemic infections poses critical threats for immunocompromised patients. These patients (e.g., oncologic and hemodialyzed patients) are often exposed to tissue damage associated with long-term vascular catheterization. Prolonged tissue damage increases the probability of wound infection due to asymptomatic *S. aureus* carriage. In this sense, it should be emphasized that *S. aureus,* as a pathogen of an outstanding clinical significance, is considered to possess a combination of such virulence factors. Special interest is directed to a triterpenoid carotenoid pigment to which the bacterium owes its major physical attribute, and subsequently the species name—a golden colony color.

Staphyloxanthin, firstly identified in 1981, is not only a carotenoid pigment but also an important virulence factor [1]. Staphyloxanthin production improves *S. aureus* cells resistance to environmental factors such as UV radiation and reactive oxygen species [2,3,4,5,6]. The exact influence of staphyloxanthin deficiency on pathogen-host interactions has been extensively studied. However, there are several reports that indicate improved immune response and improved clinical course of staphylococcal infections associated with decreased staphyloxanthin synthesis [7,8]. Staphyloxanthin is an important factor that affects the human immune response, first by decreasing the phagocytosis efficiency, second by cell protection against free oxygen radicals acting inside the phagocyte cells.

Phagocytosis represents one of the primary effector mechanisms within the innate immune response. This process, oriented on the elimination of extracellular factors (including bacterial cells), poses one of the critical points of antibacterial immunity. Efficient enhancement of the phagocytosis by either innate or adaptive immunity-derived factors (complement components and the antibodies), remains crucially dependent on direct interaction between phagocytic cells and extracellular pathogens. Unfortunately, phagocytosis depends not only on direct host-pathogen interaction but is also shaped by pathogen-dependent virulence factors such as staphyloxanthin [9,10]. Pathogens can launch a range of mechanisms to control or even evade ingestion or digestion by phagocytes. However, differentiated abilities of *S. aureus* cells to interact with the human host reach growing interest [11]. Plant-derived essential oils (EOs) and essential oil compounds (EOCs) seem promising in in the fight and prevention of staphylococcal infections, among these, in particular, fennel essential oil (FEO). It was reported that FEO addition to the *S. aureus* culture medium decrease staphyloxanthin production and deprive its colonies with golden pigmentation [12]. Furthermore, it was shown that farnesol, a compound naturally found in plant metabolite (e.g., citronella EO), completely inhibited the staphyloxanthin biochemical pathway [13]. Several researchers have also revealed that EOs can act as immune enhancers. As recent studies have shown, virgin coconut oil inhibited growth of *S. aureus* and increased the ability of the phagocytic immune cells (macrophages) [14]. It has also been proven that EOs (nutmeg, clove, niaouli, tea tree, bay laurel, lemon, red thyme, ginger), as well as EOCs (eugenol, carvacrol, bornyl acetate, isobornyl acetate) can cause mild to moderate inhibition of phagocytosis (21–40%) [15].

*trans*-Anethole (1-methoxy-4-((*E*)-propenyl)-benzene), a terpenoid formed in plants as a by-product of terpene biosynthesis, represents the major constituent of fennel, anise or star anise EOs. The acute LD_50_ (lethal dose; 50%) of *trans*-anethole was reported as > 5000 mg/kg in rabbits [16]. Due to confirmed safety of the *trans*-anethole (genotoxic or carcinogenic activity at low doses was not detected), this compound can boast about safety certification granted by the Food and Drug Administration (FDA) [17]. *trans*-Anethole is commonly used as an additive in food and pharmaceutical products. Especially due to unique taste and aroma, *trans*-anethole is primarily used in different kinds of food, such as candies, ice cream, chewing gums, and alcoholic beverages [18,19]. Additionally, according to the literature, *trans*-anethole presents antibacterial, antifungal, insecticidal, larvicidal, antioxidant, anti-inflammatory, antihypernociceptive, immunomodulatory, antitumor, cardiovascular, antithrombotic, antidiabetic, anxiolytic, local anesthetic, gastroprotective, and wound-healing properties [20,21,22,23,24,25,26].

Regarding the role of staphyloxanthin in shaping phagocytosis of staphylococci as well as the promising observations on *trans*-anethole anti-staphyloxanthin and antimicrobial effects, it has been hypothesized that *trans*-anethole can influence the staphylococcal susceptibility to phagocytosis, e.g., by interfering with staphyloxanthin production.

The primary aim of the current study was to analyze the morphological and functional changes in *trans*-anethole exposed *S. aureus*. In this context, special attention was paid to the following parameters: staphyloxanthin production, antioxidant activities, bacterial cell size. Furthermore, the study also aimed to investigate the consequences of the modifications mentioned above on the activity of human phagocytes in vitro. For this purpose, the in vitro model of staphylococcal sepsis in the human whole blood samples was performed. The following parameters were analyzed: ingestion and intracellular killing in the phagocytes and IL-8 production, recognized as the major chemoattractant. To the authors’ best knowledge, this is the first study that implements such a complex approach towards the possible impact of naturally derived substances on host-pathogen interaction.

## 2. Results

### 2.1. The Antimicrobial Activity of trans-Anethole Against S. aureus Newman Strain

The investigation showed that *trans*-anethole in the concentration of 49.4 mg/mL (MIC_50_—minimum inhibitory concentration required to inhibit the growth of 50% of microorganisms) presented the subinhibiting activity against *S. aureus* Newman strain. Futhermore, Tween 80 (at final concentration 1%) into Mueller-Hinton agar (MHA) had no impact on bacterial growth inhibition.

### 2.2. Measurement of Antioxidant Activities

The results of antioxidant activities ((represented by 2,2-diphenyl-1-picrylhydrazyl (DPPH) free radical scavenging activity, reducing power (RP), and total polyphenolic content (TPC)) of the *S. aureus* Newman cells isolated from different growth media (A–C) presents Table 1. A significant decrease (*p* < 0.05) of the DPPH scavenging activity, RP, and TPC of *S. aureus* cultured on medium C was observed in comparison to the control (medium A). Moreover, significantly higher (*p* < 0.05) DPPH scavenging activity and RP of *S. aureus* cells incubated on medium B when compared to the control (medium A) was noticed.

### 2.3. Pigment Measurements

**Figure 1:**
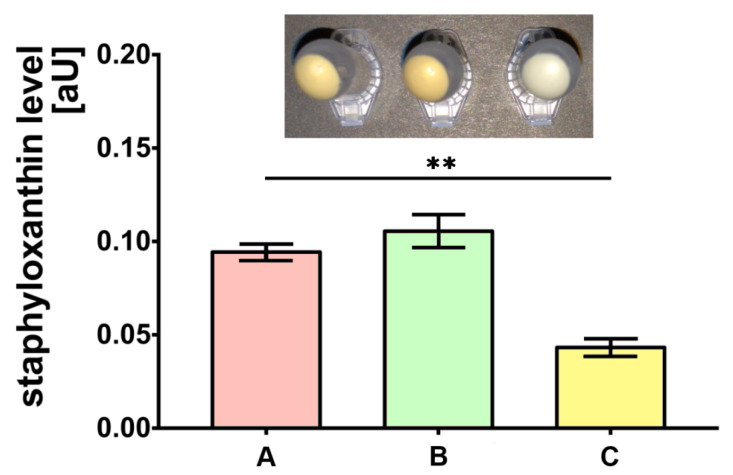
Staphyloxanthin level measurements produced by *S. aureus* Newman strain isolated from different modifications of the Mueller-Hinton agar: non-supplemented (control medium A); supplemented with 1% (*v/v*) Tween 80 (medium B); supplemented with 1% (*v/v*) Tween 80 and *trans*-anethole at subinhibitory concentration—MIC_50_ (medium C). Data are shown as the mean ± SD. ** *p* < 0.01.

The bacteria cultured on medium C were characterized by the almost two-fold lower level of staphyloxanthin production compared to the control (medium A) (*p* < 0.01). Simultaneously, the production of staphyloxanthin by bacteria cultured on medium B was not significantly different when compared to bacteria on medium A (Figure 1).

### 2.4. Analysis of Cells Size

**Figure 2:**
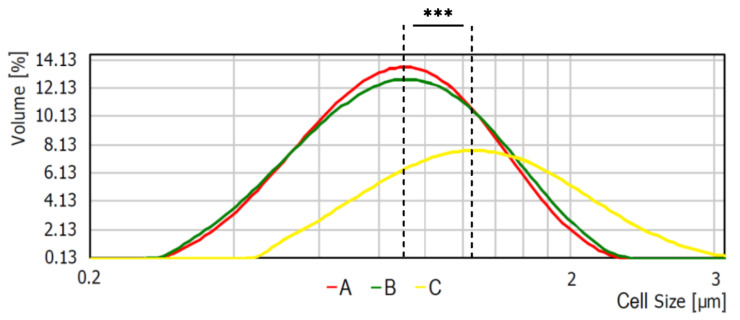
Average bacterial cell diameter of *S. aureus* Newman strain isolated from different modifications of the Mueller-Hinton agar: non-supplemented (control medium A); supplemented with 1% (*v/v*) Tween 80 (medium B); supplemented with 1% (*v/v*) Tween 80 and *trans*-anethole at subinhibitory concentration—MIC_50_ (medium C). *** *p* < 0.001.

**Figure 3:**
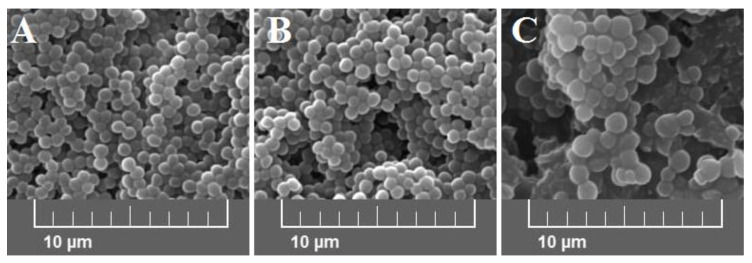
Scanning electron microscope (SEM) images at 5.00 kx of *S. aureus* Newman strain isolated from different modifications of the Mueller-Hinton agar: non-supplemented (control medium **A**); supplemented with 1% (*v/v*) Tween 80 (medium **B**); supplemented with 1% (*v/v*) Tween 80 and *trans*-anethole at subinhibitory concentration—MIC_50_ (medium **C**).

Average bacterial cells diameter for particular media were indicated as follows: medium A—0.620 ± 0.166 μm, medium B—0.619 ± 0.193 μm, and medium C—1.438 ± 0.154 μm (Figure 2). As shown, the diameter of bacterial cells cultured on medium C is significantly higher (*p* < 0.001) than bacteria cultured on the other media. The observed differences were further confirmed with the scanning electron microscope (SEM) analyses (Figure 3).

### 2.5. Fourier Transform Infrared (FTIR) Spectroscopy Analysis

**Figure 4:**
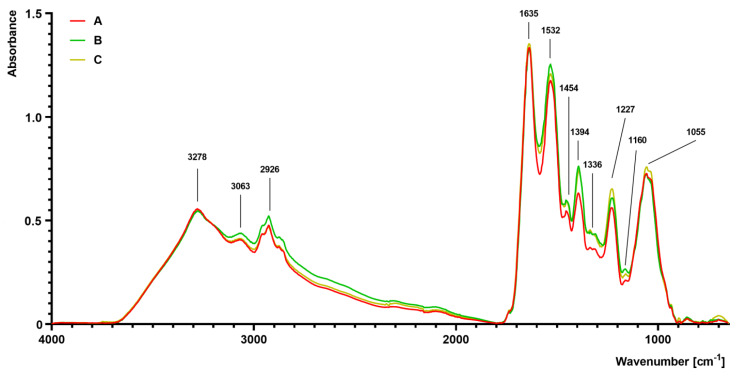
Fourier transform infrared (FTIR) spectroscopy analysis of *S. aureus* Newman strain isolated from different modifications of the Mueller-Hinton agar: non-supplemented (control medium A); supplemented with 1% (*v/v*) Tween 80 (medium B); supplemented with 1% (*v/v*) Tween 80 and *trans*-anethole at subinhibitory concentration—MIC_50_ (medium C).

The FTIR spectra of *S. aureus* grown on different media (A–C) are presented in Figure 4. The differences were observed, particularly in the complex spectral regions at 1700–1200 cm^−1^ and 1200–900 cm^−1^. The absorbance value of the sample isolated from medium B and C was slightly increased at 1454 cm^−1^ and 1394 cm^−1^ in comparison to the control sample isolated from medium A. At 1227 cm^−1^, there was a noticeable increase in absorbance in the sample isolated from medium C, followed by the medium B sample. At 1055 cm^−1^, an increased absorbance in the sample isolated from medium C was noticed. Moreover, a noticeable increase in absorbance at bands 2961 cm^−1^ (medium B) and 2926 cm^−1^ (medium B) was observed in comparison to the control sample (medium A).

### 2.6. Complete Blood Count (CBC) Analysis

CBC examination outcomes in all volunteers were identified within a normal range, and this referred to white blood cell populations, red blood cell parameters, and platelets. The results for CBC analysis are listed in Supplementary Materials Table S1.

### 2.7. Killing Assay

**Figure 5:**
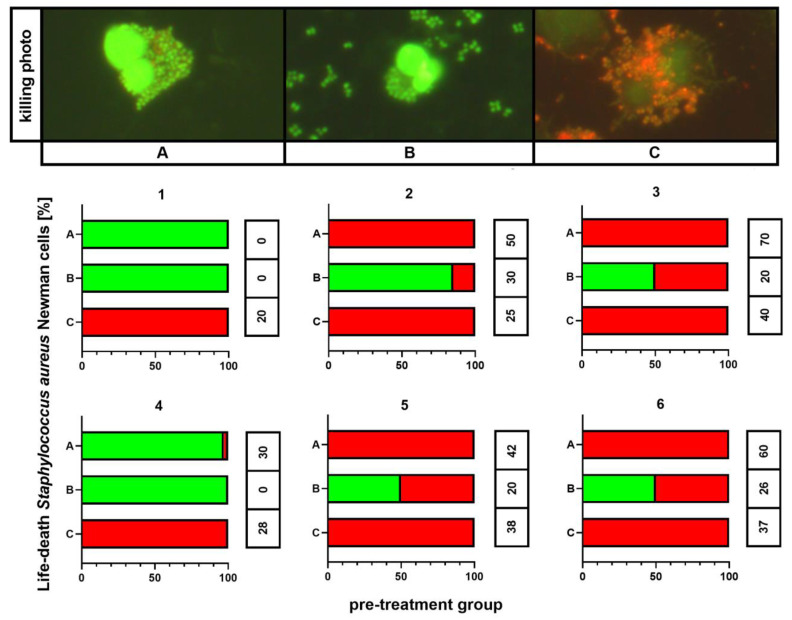
Killing activity assessment of *S. aureus* Newman cells. A—Mueller-Hinton agar non-supplemented (MHA, control), B—MHA supplemented with 1% (*v/v*) Tween 80, and C—MHA supplemented with 1% (*v/v*) Tween 80 and *trans*-anethole at subinhibitory concentration (MIC_50_). The number above each plot represents the volunteer number. The values in the table represent the killing index. Photographic evidence of intracellular killed and living bacteria is presented in the pictures above the graphs (magnification: 400x).

The outcomes of killing activity assessment are shown in Figure 5. In all individuals, 100% of phagocytes that ingested any bacteria revealed killed bacterial cells exclusively if these were cultured on medium C. This phenomenon was observed independently on the results for the control (medium A). However, the killing index (KI) appeared significantly higher (*p* < 0.001) for bacteria cultured on medium A than for these incubated on medium C: 54 ± 9 vs. 29 ± 9, respectively. Smaller percentages of killed bacteria containing phagocytes, as well as smaller KI values (26 ± 8), were observed for bacteria from medium B. In other treatment patterns, both killed and live bacteria were detected inside phagocytic cells.

### 2.8. Phagocytosis Assay

**Figure 6:**
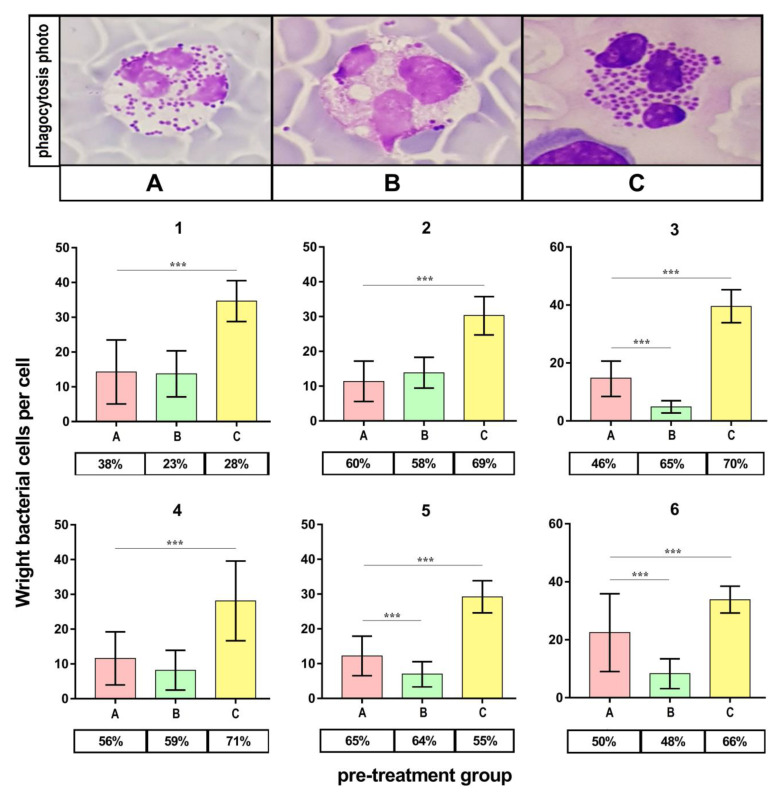
Phagocytosis assay validity of *S. aureus* Newman cells. A—Mueller-Hinton agar non-supplemented (MHA, control), B—MHA supplemented with 1% (*v/v*) Tween 80, and C—MHA supplemented with 1% (*v/v*) Tween 80 and *trans*-anethole at subinhibitory concentration (MIC_50_). The number above each plot represents the volunteer number. The values in the table represent the percentages of the phagocytic cells. Photographic evidence is presented in the pictures above the graphs (magnification: 400x). Data are shown as the mean ± SD. *** *p* < 0.001.

Figure 6 presents the outcomes of the phagocytic index (PI) evaluation. PI appeared significantly higher (*p* < 0.001) for bacteria cultured on medium C compared to the control (medium A) in all volunteers. On the contrary, the PI appeared significantly lower (*p* < 0.001) for bacteria cultured on medium B in half of the volunteers.

### 2.9. Nitroblue Tetrazolium (NBT) Dye Reduction Analysis

**Figure 7:**
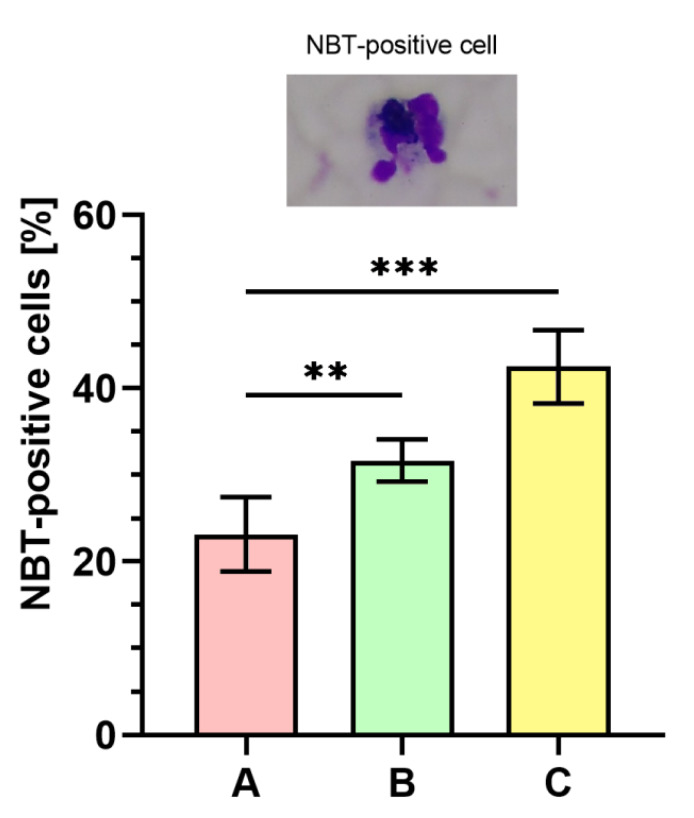
Percentages of nitroblue tetrazolium (NBT)-positive cells. A—Mueller-Hinton agar non-supplemented (MHA, control), B—MHA supplemented with 1% (*v/v*) Tween 80, and C—MHA supplemented with 1% (*v/v*) Tween 80 and *trans*-anethole at subinhibitory concentration (MIC_50_). Photographic evidence is presented in the pictures above the graph (magnification: 400x). Data are shown as the mean ± SD. ** *p* < 0.01, *** *p* < 0.001.

Figure 7 displays the outcomes of respiratory burst evaluated with NBT assay. The percentage of NBT-positive cells was significantly higher (*p* < 0.001) in phagocytes stimulated with bacteria cultured on medium C ranging between 39–50% (43 ± 4%) as compared to the bacteria from the control (medium A, 15–26%; 23 ± 4%). The percentage of NBT-positive cells was also significantly higher (*p* < 0.01) in the case of bacteria harvested from medium B (29–35%; 32 ± 2%) than in control (medium A).

### 2.10. The Influence of S. aureus Preincubated with the Subinhibitory Concentration of trans-Anethole on the Production of Interleukin-8 (IL-8)

**Figure 8:**
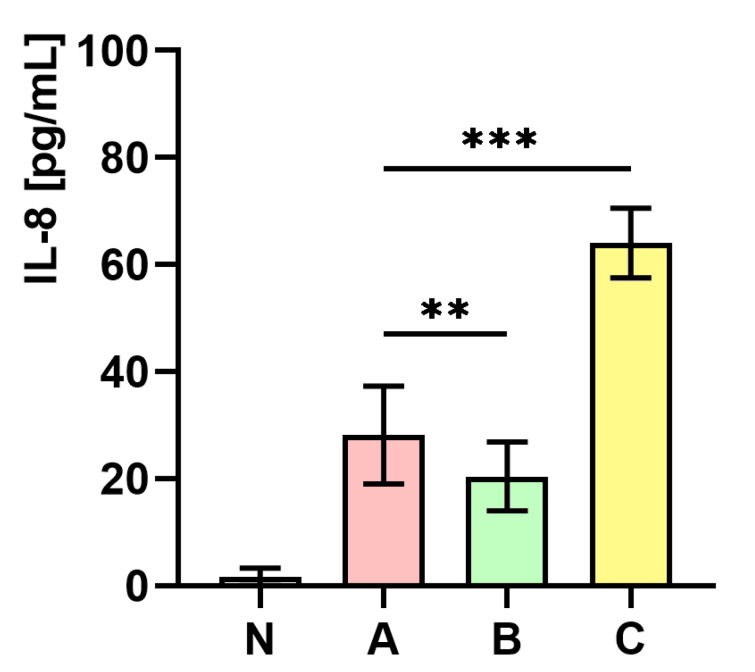
Measurements of IL-8 concentration in whole human blood non-infected (N) and infected with *S. aureus* Newman strain isolated from different modifications of the Mueller-Hinton agar: non-supplemented (control—medium A); supplemented with 1% (*v/v*) Tween 80 (medium B); supplemented with 1% (*v/v*) Tween 80 and *trans*-anethole at subinhibitory concentration—MIC_50_ (medium C). Data are shown as the mean ± SD. ** *p* < 0.01, *** *p* < 0.001.

Figure 8 presents the measurements of IL-8 concentration in whole human blood. As the figure shows, the level of IL-8 concentration in whole human blood infected with *S. aureus* cultured on medium C was almost two-fold higher (*p* < 0.001) in comparison to the control (medium A). In contrast, a significantly lower (*p* < 0.01) level of IL-8 in whole human blood infected with *S. aureus* cultured on medium B in comparison to the control (medium A) was observed.

## 3. Discussion

The present study aimed to analyze morphological and functional changes in *S. aureus* due to *trans*-anethole exposure and their consequences for phagocytic activity of human neutrophils. Cell morphology and presence of particular chemical moieties in *S. aureus* Newman strain were performed with SEM and FTIR spectroscopy, respectively. The bacteria treated with *trans*-anethole represented enlarged size and higher bands in 1227 cm^−1^ and 1055 cm^−1^ region of FTIR analysis. Functionally, these expressed smaller staphyloxanthin production and decreased antioxidant activities.

Phagocytosis efficiency was assessed on the basis of the phagocytic index, percentage of phagocytic cells, which reflects ingestion intensity, killing index, and NBT reduction reflecting intracellular killing potential. Furthermore, in order to assess the chemotactic potential of inflammatory cells, the measurements of IL-8 concentration in whole human blood samples were also included in the study. Increased values of phagocytic index, number of NBT-positive cells, and IL-8 secretion were observed in the samples stimulated with *trans*-anethole-treated *S. aureus.*

EOs and their major compounds have been gaining the growing interest among researchers worldwide. This is mostly in the context of their antimicrobial potential in the post-antibiotic era. One of the best-known examples of the successful use of EOs in treatment of bacterial infections is the tea tree oil (TTO). Edmondson et al. [27] showed that TTO can be used as an effective decolonization agent against methicillin-resistant *S. aureus* (MRSA) causing acute and chronic wounds of different etiology. As reported by those authors, when 3% solution of TTO was applied directly on the infected site, no MRSA was isolated from the swabs. Moreover, Riella et al. [28] in their studies on animal models, showed that EO obtained from *Lippia gracilis* whose main component is thymol, can be used in wound healing. Narayanan et al. [29] reported on the possibility of use of thymol and eucalyptol, as additives to the mouthwash (patent number: WO2012018519A1). Based on the aforementioned studies, we assumed that also *trans*-anethole can find similar applications. In 2017, the Australian Government Therapeutic Goods Administration (TGA) allowed to incorporate *trans*-anethole into different cosmetic and domestic products at a concentration not exceeding 10% [30]. Consequently, *trans*-anethole concentration used in our study was 49.4 mg/mL (about 5%); hence, this concentration was within the scope of the permit.

Several authors have investigated the influence of EOs (and their major compounds) on innate immunity. Özek et al. [31] indicated that the *Ferula iliensis* EO stimulated reactive oxygen species production in human neutrophils and the murine bone marrow phagocytes activation. Interesting findings have been presented in the study by Serafino et al. [32] concerning the impact of eucalyptus essential oil (EEO) on innate immunity. The authors observed the increased phagocytic activity of monocyte-derived macrophages treated with EEO, which was confirmed by morphological as well as ultrastructural modifications of these cells. Moreover, the influence on the cytokines production was differentiated. While no influence was found in terms of IL-2 and interferon gamma (IFN-γ) production, the release of proinflammatory cytokines such as IL-6, tumor necrosis factor alpha (TNF-α), and IL-4 was significantly decreased. Silva-Comar et al. [33] revealed a differentiated effect of estragole (the major compound of many EOs) on inflammatory reaction. The authors showed increased chemotactic activity of inflammatory cells, whereas the production of nitric oxide in macrophages was stimulated only within a tight dosage window (10 μg/mL).

In the current study, increased phagocytic activity, including to chemotactic activity, ingestion, and intracellular killing in the presence of *S. aureus* cells preincubated with *trans*-anethole was presented. To the authors’ best knowledge, this is the first study that implements a complex approach towards the possible impact of naturally derived substances on host-pathogen interaction.

The FTIR analyses performed in the present study showed that the higher intensity bands were indicated in the 1227 cm^−1^ and 1055 cm^−1^ spectral regions in the sample isolated from the medium that contains 1% Tween 80 and *trans*-anethole at the subinhibitory concentration (MIC_50_). It has been proven that the 1055 cm^−1^ band attributed to OH stretching coupled with CO bending and stretching of polysaccharides capsule and peptidoglycan [34]. According to Güler et al. [35], the numbers of PO_2_^-^ stretching vibrations located in a wide range ~1190–1265 cm^−1^ and around 1100–1050 cm^−1^ and their exact frequency strongly depends on the hydration state of the lipids. It can be assumed that the hydration state of the lipids in the cell wall may affect the size of the bacterial cell. Nevertheless, the exact mechanistic model in which particular components of cellular surface shape the physical properties of the bacterial cell remains to be determined.

The significance of staphyloxanthin in staphylococcal virulence has been previously reported [7]. It has been indicated that staphyloxanthin increases the antioxidant activity of staphylococcal cells. According to Pelz et al. [36], there is no impact of staphyloxanthin on the fatty acid composition of the staphylococcal cell wall. However, these authors also confirmed the influence of staphyloxanthin on cell wall modification responsible for electron and carotenoids transport. In the present study, decreased staphyloxanthin production, as well as reduced antioxidant activity, were indicated. Reduced antioxidant activity correlated with the outcomes of the intracellular killing assessment. The current study proved reduced the antioxidant activity of the bacterial cells cultivated on the medium supplemented with 1% Tween 80 and *trans*-anethole at the subinhibitory concentration (MIC_50_). On the other hand, increased content of phospholipids combined with decreased staphyloxanthin production suggests that staphyloxanthin production influence on the cell wall modification, but not via lipid components regulation.

Increased phagocytic index values revealed the higher ingestion-vulnerability of the *S. aureus* cells treated with *trans*-anethole. Additionally, *S. aureus* cells were characterized by increased peptidoglycan content as demonstrated by FTIR analysis. Peptidoglycan is recognized as a significant pathogen-associated molecular pattern (PAMPs). Thus, it seems reasonable to assume that increased peptidoglycan content in bacterial cell walls; consequently, this increased their susceptibility to phagocytes. Unfortunately, there is not enough experimental data addressing this issue in the literature on the subject. The studies on *S. aureus* phagocytosis mostly focus on adaptive immunity, including the role of specific antibodies and complement receptors in recognition of staphylococcal cells [37]. Unlike peptidoglycan, which is a non-protein diffused chemical component of the bacterial cell wall, proteins, such as the antibodies or specific receptors, can be easily detected by highly specific immunologic assays.

Production of cytokines under the influence of EOs has already been investigated by Serafino et al. [32]. The aforementioned study reported decreased production of IL-4, IL-6, and TNF-α in macrophages pretreated with EEO—unfortunately, the authors did not analyze IL-2 and IFN-γ. Consistently, Yadav and Chandra [38] observed decreased IL-1, IL-6, and TNF-α production in murine alveolar macrophages treated with EEO and its major constituent 1,8-cineole separately. The present study also indicated that secretion of the IL-8 increased after the phagocytes stimulation with *trans*-anethole-treated *S. aureus* cells. This appears remarkable in the context of the other findings, especially concerning an increased amount of peptidoglycan compound. Peptidoglycan as the PAMP is a ligand for Toll-like receptor 2 (TLR2), and the IL-8 release is mediated by TLR2-dependent pathway [39,40]. Therefore, we propose a model of an indirect influence of *trans*-anethole on chemotactic activity, which is implemented via increased peptidoglycan production in the bacterial cells, increased TLR2 ligation, and consequently, increased IL-8 production.

The number of volunteers represents a limitation of this study when considering highly complex and individual character of immune response. Therefore, particular results, e.g., the killing index, which appeared higher in control group, require further investigation. Still, the majority of the parameters that we targeted revealed increased intensity of immune reaction against *trans*-anethole-pretreated *S. aureus.* This is also true for samples with decreased killing index values when stimulated with control.

We have implemented a combination of different analytic methods in order to investigate the complexity of interaction between *trans*-anethole-treated *S. aureus* cells and the human blood. Pathogen properties were evaluated using a set of advanced techniques while immune response by more traditional methodology. This is due to the pioneering and functional character of the current study. Functional analysis of different immunity components is currently possible with the use of, e.g., flow cytometry. However, fixed conditions and reagents dedicated to such experiments exclude the possibility of own modifications. This mostly refers to bacterial strains provided within highly standardized kits. As a consequence, complex approach towards host-pathogen interaction (especially when considering pathogen modification) is limited to traditional methods and implies the use of whole blood model.

In our previous studies, we have presented positive interaction between *trans*-anethole and mupirocin in preventing biofilm formation and asymptomatic carriage of *S. aureus* [22]. Promising results obtained in the current investigation motivate to further studies on this compound in skin care products or in wound dressings. With regard to emerging character of staphylococcal infections, this appears outstandingly convenient to search for measures that could synergize antibiotics by stimulating local defense mechanisms. This reveals even larger significance when considering the potential of limited local staphylococcal infections to develop into bloodstream infections in the predestined immunocompromised hosts, such as hemodialyzed or cardiosurgical patients.

## 4. Materials and Methods

### 4.1. S. aureus Newman Susceptibility to trans-Anethole

#### 4.1.1. Culture Media Preparation

Culture media used for *trans*-anethole (CAS number: 4180-23-8; 99.0% purity; density: 0.988 g/mL at 25 °C; Sigma-Aldrich, Darmstadt, Germany) studies on *S. aureus* Newman (ATCC 25904) strain were prepared according to the procedure as described in our previous study with slight modification [22]. In brief, sterile 1% (*v/v*) Tween 80 (Sigma-Aldrich, Darmstadt, Germany) was added into cooled to 50 °C Mueller-Hinton agar (MHA; Sigma-Aldrich, Darmstadt, Germany) after autoclaving to enhance *trans*-anethole solubility. Then, 15 mL of the mixed medium was transferred to sterile Petri dishes (Ø = 90 mm), left to cool, and placed for 60 min at 37 °C. The following culture media were prepared: MHA—sterility control, MHA—control, MHA supplemented with 1% (*v/v*) Tween 80, MHA supplemented with 1% (*v/v*) Tween 80 and selected *trans*-anethole (0.19–98.8 mg/mL) concentration.

#### 4.1.2. Determination of MIC_50_ of *trans*-Anethole

The agar dilution method was performed according to the Clinical and Laboratory Standards Institute [41] procedure with own modification. *S. aureus* was cultivated for 18 h at 37 °C on Columbia agar with 5% sheep blood (bioMérieux, Warsaw, Poland). After incubation, three–five colonies were suspended in sterile phosphate-buffered saline (PBS, pH = 7.4) and washed three times. Then, diluted bacterial suspension was transferred onto prepared media (MHA–control, MHA supplemented with 1% (*v/v*) Tween 80, MHA supplemented with 1% (*v/v*) Tween 80 and selected *trans*-anethole (0.19–98.8 mg/mL) concentration) using a multichannel pipette (32 spots; 10^4^ CFU/spot, 3 mm spot diameter). After the inoculation, spots were left to dry at room temperature. All plates were incubated for 18 h at 37 °C. Minimum inhibitory concentration (MIC_100_) of *trans*-anethole was determined as the lowest concentration of *trans*-anethole, which completely inhibited the visible growth of *S. aureus* on the MHA plate. At this stage, subinhibitory concentration (MIC_50_–minimum inhibitory concentration required to inhibit the growth of 50% of microorganisms) of *trans*-anethole proportionally to the MIC_100_ value was calculated. The study was repeated twice using the MIC_50_ value of *trans*-anethole as described above. Experiments were performed in duplicate.

#### 4.1.3. Preparation of Ready-to-Use Culture Media

Culture media preparation was performed according to the method described in Section 4.1.1. At this stage, the following ready-to-use culture media were used for further research: MHA—control (medium A), MHA supplemented with 1% (*v/v*) Tween 80 (medium B), and MHA supplemented with 1% (*v/v*) Tween 80 and *trans*-anethole at subinhibitory concentration—MIC_50_ (medium C) (Supplementary Materials Figure S1).

### 4.2. Preparation of S. aureus Newman Suspensions

Suspensions 1A–C. Preparation of intracellular cell-free extracts from bacteria grown on A–C media was performed according to Ding et al. [42] with slight modifications (Supplementary Materials Figure S1). The bacterial cells were collected from the media surface (A–C), washed five times with PBS, resuspended in water:methanol (8:2, *v:v*; Sigma-Aldrich, Darmstadt, Germany) solution, and adjusted to the same concentration equivalent to no. 2 of the McFarland scale (approximately 6.0 × 10^8^ CFU/mL) using Densilameter II (Erba Diagnostic Mannheim GmbH, Mannheim, Germany). The extracts underwent 1-min ice bath ultrasonic cell disruption in a 500 mL beaker filled with small ice cubes. After removing the cell debris by centrifugation (3000× *g*, 10 min, 4 °C), the supernatant was obtained as the intracellular cell-free extracts of *S. aureus* cells.

Suspensions 2A-C. *S. aureus* Newman was cultivated for 18 h at 37 °C on Columbia agar with 5% sheep blood. After 18 h of growth, two–three colonies were harvested using the bacteriological loop, suspended in sterile PBS and adjusted to the concentration equivalent to no. 0.5 of the McFarland scale (approximately 1.5 × 10^8^ CFU/mL) using Densilameter II (Erba Diagnostic Mannheim GmbH, Mannheim, Germany). Then, 100 µL of staphylococcal suspension in PBS was introduced on the A-C media surface, spread using a spreader, and re-incubated at 37 °C for 18 h. After that stage, five–seven colonies were transferred into 1 mL of PBS, vortexed for 2 min and centrifuged (3000× *g* for 10 min). The washing procedure was repeated five times. Finally, the washed pellet was suspended in 1 mL of PBS and used immediately before each experiment (Supplementary Materials Figure S1).

### 4.3. Determination of Antioxidant Capacity of S. aureus Cells Treated with trans-Anethole

#### 4.3.1. Determination of 2,2-diphenyl-1-picrylhydrazyl (DPPH) Free Radical Scavenging Activity

The DPPH radical-scavenging capacity of *S. aureus* samples was determined, according to Ding et al. [42]. Briefly, 1 mL of suspension (1A–C) was mixed with 2 mL of methanolic DPPH radical (0.05 mM; Sigma-Aldrich, Darmstadt, Germany) solution, stirred vigorously, and incubated in the dark at room temperature for 30 min. The absorbance of the resulting solution was measured at 517 nm using a spectrophotometer (Evolution 220 UV-Visible Spectrophotometer, Thermo Fisher Scientific, Waltham, MA, USA). A mixture of DPPH and water:methanol (8:2, *v:v*) was used as a control sample. Experiments were performed in triplicate. The scavenging activity was calculated according to Equation (1):

(1) $$\mathrm{DPPH}\;\mathrm{free}\;\mathrm{radical}\;\mathrm{scavenging}\;\mathrm{activity}\;\left(\%\right)=\frac{A_{\mathrm{control}}{\;-\;A}_{\mathrm{sample}}}{A_{\mathrm{control}}}\times 100$$

#### 4.3.2. Reducing Power (RP) Assay

RP of *S. aureus* samples was determined according to the method reported by Ding et al. [42], with slight modification as described by Łopusiewicz et al. [43]. 0.5 mL of the suspension (1A–C) was mixed with 0.5 mL of water:methanol (8:2, *v:v*) solution and 0.5 mL of 1% potassium ferricyanide (Sigma-Aldrich, Darmstadt, Germany) and incubated at 50 °C for 20 min. After cooling, 1 mL of this mixture was transferred to 1 mL of 0.1% ferric chloride (Sigma-Aldrich, Darmstadt, Germany), and the absorbance was measured at 700 nm using a spectrophotometer (Evolution 220 UV-Visible spectrophotometer, Thermo Fisher Scientific Inc., Waltham, DE, USA). Experiments were performed in triplicate. The RP was calculated according to Equation (2):

(2) $$\mathrm{Reducing}\;\mathrm{power}\;\left(\%\right)=\frac{A_{\mathrm{control}}{\;-\;A}_{\mathrm{sample}}}{A_{\mathrm{control}}}\times 100$$

#### 4.3.3. Determination of Total Polyphenolic Content (TPC)

The total polyphenolic content (TPC) in *S. aureus* Newman cultures was determined using Folin-Ciocalteu reaction according to Łopusiewicz et al. [43] with slight modification. 20 μL of suspension (1A–C) was mixed with 1.5 mL of deionized water and 100 μL of the Folin-Ciocalteu reagent (Sigma-Aldrich, Darmstadt, Germany), stirred gently for 5 min and mixed with 300 μL of a saturated solution of sodium carbonate (Sigma-Aldrich, Darmstadt, Germany). The mixture was allowed to stand in darkness for 30 min at 40 °C, followed by an absorbance reading at 765 nm using a spectrophotometer (Evolution 220 UV-Visible spectrophotometer, Thermo Fisher Scientific Inc., Waltham, DE, USA). A calibration curve of gallic acid (Sigma-Aldrich, Germany) in water:methanol (1:1, *v:v*) (0, 50, 100, 200, 400 and 500 μg/mL) was prepared and TPC was calculated as milligrams of gallic acid equivalents (GAE)/gram of *S. aureus* cell dry mass (mg GAE/g DM). Experiments were performed in triplicate.

### 4.4. Staphyloxanthin Production Assessment

Staphyloxanthin extraction was performed as described by Song et al. [7] with minor modification. Suspensions (2A–C) were adjusted to the concentration equivalent to no. 4 of the McFarland scale (approximately 12.0 × 10^8^ CFU/mL) and centrifuged (3000× *g*, 4 °C, 10 min). Then, pellets were suspended in 100 µL of 99.8% methanol (Sigma-Aldrich, Darmstadt, Germany) and Eppendorf tubes were placed in a thermoblock at 55 °C for 20 min. Next, the samples were centrifuged (3000× *g*, 4 °C, 10 min), and the absorbance of the supernatant was measured at 462 nm wavelength using a microplate reader (EnVision Multilabel Plate Reader 2105, Perkin Elmer, Shelton, CT, USA). Experiments were performed in triplicate.

### 4.5. Staphylococcal Cell Analysis Under trans-Anethole Pressure

#### 4.5.1. Measurements of the Particle Size Distribution

The bacterial cells size distribution (from colonies grown on different microbiological media: A–C) was analyzed by the use of particle size analyzer Mastersizer 2000 (Malvern Panalytical Ltd., Malvern, United Kingdom) as described in our previous study [44]. Briefly, suspensions (2A–C) were centrifuged (3000× *g* for 10 min), and suspended in PBS with 0.01% (*v:v*) surfactant–sodium dodecyl sulfate (Sigma-Aldrich, Germany) solution to reach concentration equivalent to no. 7 of the McFarland scale (approximately 21.0 × 10^8^ CFU/mL) and dispersed in 800 mL (stirrer speed—2000 rpm) of distilled water at room temperature to reach obscurance in the range 5%. Each sample was measured in triplicate.

#### 4.5.2. Scanning Electron Microscopy (SEM)

The SEM analyses of bacteria grown on different culture media (A–C) were performed according to the method described by Xu et al. [45] with a few modifications as described in our previous work [44]. In brief, suspensions (2A–C) were adjusted to the concentration equivalent to no. 0.5 of the McFarland scale, centrifuged (3000× *g* for 10 min), fixed with glutaraldehyde (2.5%, *v/v*; Chempur, Piekary Slaskie, Poland), and dehydrated in a gradient ranging ethanol (30–100%, *v/v*; Chempur, Poland). After this, the ethanol was replaced with tert-butanol (Chempur, Poland) at room temperature. The staphylococcal samples were dried for 8 h, and coated with gold for 90 s. Bacterial cells (the average size of bacterial cells) were observed using the scanning electron microscope (Vega 3 LMU, Tescan, Brno, Czech Republic) and analyzed using Tescan Essence^TM^ software (Tescan, Brno, Czechia).

#### 4.5.3. Determination of Functional Groups in Staphylococcal Cells by the Use of Fourier Transform Infrared (FTIR) Spectroscopy

In order to confirm the presence of particular chemical moieties in staphylococcal cells incubated on different culture media (A–C), FTIR spectroscopy analyses were performed as described previously [44]. In brief, suspensions (2A–C) were adjusted to the concentration equivalent to no. 4 of the McFarland scale, centrifuged (3000× *g* for 10 min), dried, and scanned at a range between 650 cm^−1^ and 4000 cm^−1^ (64 scans and 1 cm^−1^ resolution). The obtained spectra were normalized, baseline corrected, and analyzed using Spectrum^TM^ software (v10, Perkin Elmer Spectrophotometer, Waltham, MA, USA).

### 4.6. Whole Blood Model of Staphylococcal Sepsis

The study was conducted on the whole blood samples obtained from healthy volunteers (*n* = 6). According to the principles set by the Declaration of Helsinki, each healthy volunteer signed informed consent before participating in the study. The study was approved by the Ethics Committee of the Pomeranian Medical University of Szczecin (approval number: KB-0012/86/18). Fresh human whole blood from volunteers (the population sex ratio—50%:50%; age—20–40; Caucasian ethnic group; without either concomitant chronic disorders or infection conditions requiring antibiotic therapy in a month prior to the experiment) was collected in S-Monovette (Sarstedt, Nümbrecht, Germany) tubes. Complete blood count (CBC) on Sysmex XN-2000 (Sysmex, Kobe, Japan) apparatus was examined in all.

The whole blood model of staphylococcal sepsis was designed as multiple experiments at a one-time point (Supplementary Materials Figure S1). Suspensions (2A–C) were added to fresh human blood. Subsequently, the following assays were performed as described below: killing, phagocytosis, stimulated nitroblue tetrazolium (NBT) dye reduction, and IL-8 production assessment.

#### 4.6.1. Killing Assay

The killing assay was performed according to Krumholz et al. [46] with own modification. In the procedure, 1 mL of suspension (2A–C) was adjusted to the concentration equivalent to no. 2 of the McFarland scale and immediately added to fresh human blood in the S-Monovette tube. The sample was gently mixed by inversion. The ratio of whole blood to bacterial suspension was 3:1 (*v:v*). After mixing, 0.5 mL of blood was placed on a coverslip and incubated for 30 min in a humidified atmosphere at 37 °C. After 30 min incubation, the blood clots were removed using Hank’s balanced salt solution (HBSS, Biomed, Cracow, Poland). Subsequently, 200 µL of acridine orange (200 µg/10 mL PBS; Fluka, Buchs, Switzerland) was added between the coverslip and microscope slide. The samples were observed at 455 nm using Zeiss PrimoStar iLED fluorescence microscope (Carl Zeiss MicroImaging GmBH, Jena, Germany). Number of intracellular killed (red fluorescence) and living bacteria (green fluorescence), as well as killing index (KI; a number of killed bacteria/100 phagocytic cells) and the percentage of phagocytic cells containing killed bacteria were calculated.

#### 4.6.2. Phagocytosis Assay

Phagocytosis assay was performed as described by Lu et al. [47] with own modification. Suspensions (2A–C) were adjusted to the concentration equivalent to no. 2 of the McFarland scale and killed by incubating the microorganisms at 99 °C for 60 min. Heat-killed bacterial suspensions (0.5 mL) were washed three times using PBS, opsonized using fresh human blood in S-Monovette tubes containing EDTA, and incubated at 37 °C for 30 min with gentle rotation. The ratio of whole blood to bacterial suspension was 2:1 (*v:v*). After incubation, tubes were centrifuged (500× *g*, 4 °C, 10 min) to synchronize phagocytosis. Then, the supernatant, including phagocytes, was immediately transferred onto a microscope slides, smeared, and left to dry. The samples were stained with Giemsa dye (ANALAB, Warsaw, Poland) for 20 min and left to dry. 

The phagocytic index (PI) and phagocytosis (%) were calculated according to Equations (3) and (4), respectively [48]:

(3) $$\mathrm{Phagocytic}\;\mathrm{index}\;(\mathrm{PI})=\frac{\mathrm{Total}\;\mathrm{number}\;\mathrm{of}\;\mathrm{phagocytized}\;\mathrm{bacteria}}{\mathrm{Number}\;\mathrm{of}\;\mathrm{phagocytic}\;\mathrm{cells}\;\mathrm{phagocytizing}\;\mathrm{bacteria}}$$

(4) $$\%\;\mathrm{Phagocytosis}=\frac{\mathrm{Number}\;\mathrm{of}\;\mathrm{phagocytic}\;\mathrm{cells}\;\mathrm{phagocytizing}\;\mathrm{bacteria}}{\mathrm{Total}\;\mathrm{number}\;\mathrm{of}\;\mathrm{phagocytic}\;\mathrm{cells}\;\mathrm{counted}}\;\times \;100$$

#### 4.6.3. Stimulated Nitroblue Tetrazolium (NBT) Dye Reduction

The stimulated NBT-reduction assay was conducted according to Glasser and Fiederlein [49] with their own modification. According to the method described in Section 4.6.2., heat-killed suspensions (2A–C) (0.05 mL) were washed three times using PBS and mixed in Eppendorf tube with 100 µL of NBT (Sigma-Aldrich, Darmstadt, Germany) solution and 0.5 mL of serum, which was separated from fresh human blood by centrifugation (500× *g*, 4 °C, 10 min). The mixtures were incubated for 30 min at 37 °C with 5% (*v/v*) CO_2_ and in atmospheric humidity. After incubation, mixtures were transferred to a microscope slides, smeared, and left to dry. The slides were stained with Giemsa dye for 20 min, left to dry, and observed with the optical microscope. NBT-positive cells showed a diffusely blue cytoplasm. One hundred neutrophil cells were counted, and the percentage of NBT-positive cells was calculated.

#### 4.6.4. IL-8 Detection—ELISA Assay

Cytokine assay was performed as described by Skjeflo et al. [50] with slight modification. Suspensions (2A-C) were adjusted to the concentration equivalent to no. 4 of the McFarland scale. Then, 0.1 mL of these suspensions were added to fresh human blood (0.9 mL) in a sterile Eppendorf tube and gently mixed by inversion. Aliquots of whole blood in sterile tubes were incubated for 2 h at 37 °C with gentle rotation. After incubation, samples were centrifuged (500× *g*, 7 min, 4 °C), and obtained plasma was stored at −80 °C until further analysis. The cytokine level in plasma was detected using Human IL-8 ELISA Kit (BioVendor, Brno, Czechia) according to the manufacturers instruction. The absorbance was measured at 450 and 630 nm with a microplate reader (Perkin Elmer Lambda 650 UV/VIS, Waltham, MA, USA). Bacteria grown on medium A (non-supplemented) were used as a control. Furthermore, the measurement of IL-8 concentration in non-infected (blood + PBS) whole human blood was performed (N). All experiments were conducted in duplicate.

### 4.7. Statistical Analysis

Data were analyzed with Shapiro-Wilk for normal distribution assessment, and subsequently, an ANOVA Kruskal-Wallis test to assess the significance of differences (staphyloxanthin, phagocytosis, killing, cell size distribution, and NBT assays, as well as IL-8 detection). The one-way ANOVA, followed by NIR Fisher test, was used to check for statistically significant differences in the antioxidant tests. Statistical significance was considered with following values: * *p* < 0.05, ** *p* < 0.01, *** *p* < 0.001. Data were shown as mean ± standard deviation (SD). All statistical analyses were performed using GraphPad Prism 5.02 (GraphPad Software, San Diego, CA, USA). 

## 5. Conclusions

*trans*-Anethole treatment significantly influences the morphological and functional changes in *S. aureus* Newman strain. This was revealed by decreased antioxidant activities, decreased staphyloxanthin production as well as larger cell size. Furthermore, *trans*-anethole-treated cells were characterized by tendency to diffuse in comparison to the non-treated cells. Several cell components, such as phospholipids and peptidoglycan, were found remarkably elevated in the cultures treated with *trans*-anethole. As a result of the aforementioned cellular changes, the bacteria were phagocytized by neutrophils more efficiently—ingestion and intracellular killing of phagocytosed bacteria as well as chemotactic activity of neutrophils in the presence of *trans*-anethole modified *S. aureus* were at a higher level as compared to the control (unmodified *S. aureus*). Furthermore, increased peptidoglycan content possibly induces IL-8 production in a TLR2-dependent manner. The influence of modified bacteria appeared complex and affected different stages of phagocytosis beginning with recruitment of phagocytes, ending with intracellular killing mechanisms directed against ingested bacteria. We are aware that findings presented in this work are of in vitro character and require thorough testing in animal models before clinical trials may be performed. However, it should be emphasized that the application of *trans*-anethole for purpose of *S. aureus*, including MRSA treatment can be one of the most promising directions in fighting infection caused by this microorganism. Bearing this in mind, our results suggest that *trans*-anethole represents a promising measure in combating severe staphylococcal infections, which has important translational potential for clinical applications.

## Figures and Tables

**Table 1:** Antioxidant activities of *S. aureus* Newman strain isolated from different modifications of the Mueller-Hinton agar: non-supplemented (control medium A); supplemented with 1% (*v/v*) Tween 80 (medium B); supplemented with 1% (*v/v*) Tween 80 and *trans*-anethole at subinhibitory concentration—MIC_50_ (medium C).

Medium	DPPH ^1^ Free Radical scavenging Activity (%)	Total Polyphenolic Content (mg GAE/g DM ^2^)	Reducing Power (%)
A	16.60 ± 0.24 ^b^	0.0158 ± 0.000 ^a^	3.37 ± 1.35 ^b^
B	27.52 ± 0.15 ^a^	0.0141 ± 0.005 ^b^	6.96 ± 1.11 ^a^
C	6.09 ± 0.83 ^c^	0.0101 ± 0.002 ^c^	2.73 ± 1.48 ^c^

^1^ DPPH—2,2-diphenyl-1-picrylhydrazyl. ^2^ mg GAE/g DM—milligrams of gallic acid equivalents/gram of *S. aureus* cell dry mass.Values are means ± SD of triplicate determinations. Means with different superscript letters (a–c) in the same column are significantly different at *p* < 0.05.

## Supplementary Materials

Supplementary materials can be found at https://www.mdpi.com/1422-0067/21/11/4178/s1. Table S1: Complete blood count (CBC) analysis of volunteers. Figure S1: General scheme of research methodology.

## Abbreviations

CBC	complete blood count
DPPH	2,2-diphenyl-1-picrylhydrazyl
EOs	essential oils
EOCs	essential oil compounds
EEO	eucalyptus essential oil
FEO	fennel essential oil
KI	killing index
MRSA	methicillin-resistant *Staphylococcus aureus*
PI	phagocytic index
RP	reducing power
TPC	total polyphenolic content
TTO	tea tree oil
